# Copper Oxide Nanoparticles Induced Mitochondria Mediated Apoptosis in Human Hepatocarcinoma Cells

**DOI:** 10.1371/journal.pone.0069534

**Published:** 2013-08-05

**Authors:** Maqsood A. Siddiqui, Hisham A. Alhadlaq, Javed Ahmad, Abdulaziz A. Al-Khedhairy, Javed Musarrat, Maqusood Ahamed

**Affiliations:** 1 Department of Zoology, College of Science, King Saud University, Riyadh, Saudi Arabia; 2 King Abdullah Institute for Nanotechnology, King Saud University, Riyadh, Saudi Arabia; 3 Department of Physics and Astronomy, College of Science, King Saud University, Riyadh, Saudi Arabia; 4 Department of Agricultural Microbiology, Faculty of Agricultural Sciences, Aligarh Muslim University, Aligarh, India; Indian Institute of Toxicology Research, India

## Abstract

Copper oxide nanoparticles (CuO NPs) are heavily utilized in semiconductor devices, gas sensor, batteries, solar energy converter, microelectronics and heat transfer fluids. It has been reported that liver is one of the target organs for nanoparticles after they gain entry into the body through any of the possible routes. Recent studies have shown cytotoxic response of CuO NPs in liver cells. However, the underlying mechanism of apoptosis in liver cells due to CuO NPs exposure is largely lacking. We explored the possible mechanisms of apoptosis induced by CuO NPs in human hepatocellular carcinoma HepG2 cells. Prepared CuO NPs were spherical in shape with a smooth surface and had an average diameter of 22 nm. CuO NPs (concentration range 2–50 µg/ml) were found to induce cytotoxicity in HepG2 cells in dose-dependent manner, which was likely to be mediated through reactive oxygen species generation and oxidative stress. Tumor suppressor gene p53 and apoptotic gene caspase-3 were up-regulated due to CuO NPs exposure. Decrease in mitochondrial membrane potential with a concomitant increase in the gene expression of bax/bcl2 ratio suggested that mitochondria mediated pathway involved in CuO NPs induced apoptosis. This study has provided valuable insights into the possible mechanism of apoptosis caused by CuO NPs at *in vitro* level. Underlying mechanism(s) of apoptosis due to CuO NPs exposure should be further invested at *in vivo* level.

## Introduction

Copper oxide nanoparticles (CuO NPs) are used as industrial catalysts in manufacturing processes and are heavily utilized in semiconductor devices, gas sensor, batteries, solar energy converter, microelectronics and heat transfer fluids [1], [2]. Due to their antimicrobial properties CuO NPs are also employed in textiles, paints, plastics and food containers [3], [4]. The increasing production of CuO NPs has led to major concerns regarding the potential toxicity to the environment and human health. CuO NPs have been shown to be toxic to aquatic organisms such as freshwater shredder, juvenile carp, mussels and ciliated protozoa [5]–[8]. Recent studies have shown the oxidative stress mediated toxicity of CuO NPs in different human cell lines e.g. human lung epithelial (A549), human cardiac microvascular endothelial, kidney and neuronal cells [9]–[13]. It is also reported in the scientific literature that liver is one of the target organs for nanoparticles after they gain entry into the body through any of the possible routes [14], [15]. There are very limited studies evaluating the toxicity of CuO NPs in human liver cell lines. Wang et al [16] (2011) observed that cytoxicity of CuO NPs in HepG2 cells and catfish primary hepatocytes was mediated through ROS. Recently, Piret et al [17] (2012) found that CuO NPs induce ROS mediated cytotoxicity and pro-inflammatory response in HepG2 cells. However, the underlying mechanism of apoptosis in HepG2 cells due to CuO NPs exposure is largely lacking.

Apoptotic cell death is a key process in cancer development and progression. The ability of cancer cells to avoid apoptosis and continue to propagate is one of the basic characteristics of cancer [18]. There are several genes that involved in apoptotic cell death. The p53 protein is regarded as the master guardian of the cell is able to activate cell cycle checkpoints, DNA repair and apoptosis response to maintain genomic stability [19]. In the presence of DNA damage or cellular stress, p53 protein triggers cell cycle arrest to provide time for the damage to be repaired or self-mediated apoptosis [20], [21]. The bcl-2 protein has an anti-apoptotic effect, whereas the bax is known for pro-apoptotic activity [22]. The ratio of bax/bcl-2 protein represents a cell death switch, which determines the life or death of cells in response to an apoptotic stimulus; an increased bax/bcl-2 ratio decreases the cellular resistance to apoptotic stimuli, leading to apoptosis [23], [24]. It is also reported that destabilization of the mitochondrial integrity by apoptotic stimuli precedes activation of caspases leading to apoptosis [25]. In view of significant lack of knowledge about the mechanisms of CuO NPs toxicity, this study was designed to investigate the possible mechanisms of apoptosis induced by CuO NPs in human hepatocellular carcinoma (HepG2) cells through ROS via mitochondrial pathway. HepG2 cell line is a classical hepatic model used to test the compounds that are potentially cytotoxic, genotoxic or affect hepatocyte functions [26], [27].

## Materials and Methods

### Reagents

Dulbecco's modified eagle's medium (DMEM), Hank's balanced salt solution (HBSS), fetal bovine serum (FBS), penicillin-streptomycin and trypsin were purchased from Invitrogen Co. (Carlsbad, CA, USA). MTT [3–(4,5–2-yl)-2,5-diphenyltetrazoliumbromide], glutathione (GSH), 5,5-dithio-bis-(2-nitrobenzoic acid) (DTNB), 2,7-dichlorofluorescin diacetate (DCFH-DA), N-acetyl-cystein (NAC), Rhodamine-123 dye (Rh123), anti-bax antibody, anti-bcl-2 antibody and anti-β-actin antibody were obtained from Sigma-Aldrich (St. Louis, MO, USA). Anti-p53 antibody and cleaved caspase-3 antibody (Asp175, 17kDa) were bought from Cell Signaling Technology, Inc. (Danver, MA, USA). Secondary antibodies and RIPA buffer were purchased from Santa Cruz Biotechnology, Inc. (Santa Cruz, CA, USA). All other chemicals used were of the highest purity available from commercial sources.

### Synthesis of CuO nanoparticles

CuO NPs were synthesized by aqueous precipitation method using copper (II) acetate [Cu (CH_3_COO)_2·_H_2_O)] (98%, Sigma-Aldrich) as a precursor and sodium hydroxide (NaOH) as a reducing agent. In brief, 0.2 M copper (II) acetate solution (600 ml) and glacial acetic acid (CH_3_COOH) (2 ml) were added into a round-bottomed flask and heated to boiling under magnetic stirring. Then, 30 ml of 6 M NaOH solution was poured into the flask. The colour of the solution turned from blue to black immediately, and a black suspension formed simultaneously. The reaction was carried out under stirring and boiling for 2.5 h. The mixture was cooled to room temperature and centrifuged. Then, a wet CuO precipitate was obtained. The precipitates were filtered, washed with distilled water and absolute ethanol for several times. The resulting product was dried (at 60°C for 6 h) to obtain the dry powder of CuO NPs.

### CuO nanoparticles characterization

The crystalline nature of CuO NPs was carried out by taking X-ray diffraction (XRD) pattern. The XRD pattern of CuO NPs was acquired at room temperature with the help of PANalytical X'Pert X-ray diffractometer equipped with a Ni filtered using Cu K_α_ (λ = 1.54056 Å) radiations as X-ray source. Morphology of CuO NPs was examined by field emission scanning electron microscope (FESEM, JSM-7600F, JEOL Inc., Japan) and field emission transmission electron microscopy (FETEM, JEM-2100F, JEOL Inc., Japan) at an accelerating voltage of 15 kV and 200 kV respectively. Energy dispersive X-ray spectroscopy (EDS) was utilized used to see the elemental composition (purity) of the prepared CuO NPs. Brunauer-Emmet-Teller (BET) surface area measurement of CuO NPs was determined by multipoint nitrogen adsorption using a Beckman Coulter SA3100 device (Beckman Coulter, Inc., CA, USA).

The average hydrodynamic size and zeta potential of CuO NPs in the water and complete cell culture medium were determined by dynamic light scattering (DLS) (Nano-Zetasizer-HT, Malvern Instruments, Malvern, UK) as described by Murdock et al [28].

### Cell Culture and CuO nanoparticles treatment

HepG2 cells were obtained from American Type Culture Collection (ATCC) (Manassas, VA, USA). Cells were used between passages 10 and 20 and cultured in MEM medium supplemented with 10% FBS, 100 U/ml penicillin-streptomycin, 1 mM sodium pyruvate and 1.5 g/l sodium bicarbonate at 5% CO_2_ and 37°C. At 85% confluence, cells were harvested using 0.25% trypsin and were sub-cultured into 75 cm^2^ flask, 6-well plate or 96-well plate according to selection of experiments. Cells were allowed to attach the surface for 24 h prior to nanoparticles exposure. CuO NPs were suspended in complete cell culture medium and diluted to appropriate concentrations (2, 5, 10, 25 and 50 µg/ml). The dilutions of CuO NPs were then sonicated using a sonicator bath at room temperature for 15 min at 40W to avoid nanoparticles agglomeration prior to administration to the cells. Selection of 2–50 µg/ml concentration of CuO NPs was based on a preliminary dose-response study (data not shown). Under some conditions, HepG2 cells were pre-exposed for 1 h with 10 mM of N-acetyl-cystein (NAC) before 24 h co-exposure with or without CuO NPs. Cells not exposed to CuO NPs served as control in each experiment.

### MTT assay

MTT assay was carried out following the procedure as described by Mossman [29] with some modifications [30]. The MTT assay assesses the mitochondrial function by measuring ability of viable cells to reduce MTT into blue formazon product. In brief, 1×10^4^ cells/well were seeded in 96-well plates and exposed to different concentrations of CuO NPs for 24 h. At the end of exposure, medium was removed from each well to avoid interference of nanoparticles and replaced with new medium containing MTT solution in an amount equal to 10% of culture volume, and incubated for 3 h at 37°C until a purple colored formazan product developed. The resulting formazan product was dissolved in acidified isopropanol. Further, the 96-well plate was centrifuged at 2300 g for 5 min to settle down the remaining nanoparticles present in the solution. Then, a 100 µl supernatant was transferred to other fresh wells of 96-well plate and absorbance was measured at 570 by using a microplate reader (Synergy-HT, BioTek).

### NRU assay

Neutral red uptake (NRU) assay was performed following the procedure as described by Borenfreund and Puerner [31] with some modifications [30]. In brief, 1×10^4^ cells/well were seeded in 96-well plates and exposed to different concentrations of CuO NPs for 24 h. At the end of exposure, test solution was aspirated and cells were washed with phosphate buffer saline (PBS) twice and incubated for 3 h in medium supplemented with neutral red (50 μg/ml). The medium was washed off rapidly with a solution containing 0.5% formaldehyde and 1% calcium chloride. Cells were further incubated for 20 min at 37°C in a mixture of acetic acid (1%) and ethanol (50%) to extract the dye. Further, 96-well plate was centrifuged at 2300 g for 5 min to settle down the remaining nanoparticles present in the solution. Then, a 100 µl supernatant was transferred to other fresh wells of 96-well plate and absorbance was measured at 540 by using a microplate reader (Synergy-HT, BioTek).

### Assay of mitochondrial membrane potential

Mitochondrial membrane potential (ΔΨ) was measured following the protocol of Zhang et al [32]. In brief, control and treated cells were harvested and washed twice with PBS. Cells were further exposed with 10 µg/ml of Rhodamine-123 fluorescent dye for 1 h at 37°C in dark. Again cells were washed twice with PBS. Then, fluorescence intensity of Rhodamine-123 was measured using upright fluorescence microscope (OLYMPUS CKX 41) by grabbing the images at 20× magnification.

### Preparation of crude cell extract

For the measurement of lipid peroxidation (LPO) and glutathione (GSH), HepG2 cells were cultured in 75-cm^2^ culture flask and exposed to CuO NPs at the concentrations of 2, 5 and 10 µg/ml for 24 h. After the treatment, cells were harvested in ice-cold phosphate buffer saline by scraping and washed with phosphate buffer saline at 4°C. The cell pellets were then lysed in cell lysis buffer [1X 20 mM Tris-HCl (pH 7.5), 150 mM NaCl, 1 mM Na_2_EDTA, 1% Triton, 2.5 mM sodium pyrophosphate] as described in our previous publication [26]. Following centrifugation (15000 g for 10 min at 4°C) the supernatant (cell extract) was maintained on ice.

### Lipid peroxidation assay

The extent of membrane LPO was estimated by measuring the formation of malondialdehyde (MDA) using the method of Ohkawa et al [33]. MDA is one of the end products of membrane LPO. Briefly, a mixture of 0.1 ml cell extract and 1.9 ml of 0.1 M sodium phosphate buffer (pH 7.4) was incubated at 37°C for 1 h. After the incubation mixture was precipitated with 5% trichloroacetic acid (TCA) and centrifuged (2300 g for 15 min at room temperature) to collect supernatant. Then 1.0 ml of 1% thiobarbituric acid (TBA) was added to the supernatant and placed in the boiling water for 15 min. After cooling to room temperature absorbance of the mixture was taken at 532 nm and was converted to MDA and expressed in nmole MDA/mg protein using molar extinction coefficient of 1.56×10^5^ M^−1^ cm^−1^. A reaction mixture devoid of cell extract served as control.

### Intracellular glutathione assay

GSH level was quantified using Ellman's method [34]. Briefly, a mixture of 0.1 ml of cell extract and 0.9 ml of 5% TCA was centrifuged (2300 g for 15 min at 4°C). Then 0.5 ml of supernatant added into 1.5 ml of 0.01% DTNB and the reaction was monitored at 412 nm. The amount of GSH was expressed in terms of nmole/mg protein.

### Measurement of intracellular reactive oxygen species generation

The production of intracellular ROS was measured using 2,7-dichlorofluorescin diacetate (DCFH-DA) as described by Wang and Joseph [35] with some modifications [36]. The DCFH-DA passively enters the cell where it reacts with ROS to form the highly fluorescent compound dichlorofluorescein (DCF). In brief, HepG2 cells (5×10^4^) were seeded in 6-well plates and allowed for adherence. Following respective exposure, cells were washed twice with PBS and incubated for 30 min in dark in culture medium (without FBS) containing DCFH-DA (20 μM). The control and treated cells were visualized by use of a fluorescence microscope (OLYMPUS CKX 41) by grabbing the images at 20× magnification.

### Total RNA isolation and quantitative real-time PCR analysis

HepG2 cells were cultured in 6-well plates and exposed to 10 µg/ml of CuO NPs for 24 h. At the end of exposure, total RNA was extracted by RNeasy mini Kit (Qiagen,Valencia, CA, USA) according to the manufacturer's instructions. Concentration of the extracted RNA were determined using Nanodrop 8000 spectrophotometer (Thermo-Scientific, Wilmington, DE) and the integrity of RNA were visualized on 1% agarose gel using gel documentation system (Universal Hood II, BioRad, Hercules, CA). The first strand cDNA was synthesized from 1 µg of total RNA by Reverse Transcriptase using M-MLV (Promega, Madison, WI) and oligo (dT) primers (Promega) according to the manufacturer's protocol. Quantitative real-time PCR (RT-PCRq) was performed by QuantiTect SYBR Green PCR kit (Qiagen) using ABI PRISM 7900HT Sequence Detection System (Applied Biosystems, Foster City, CA). Two microliter of template cDNA was added to the final volume of 20 µl of reaction mixture. Real-time PCR cycle parameters included 10 min at 95°C followed by 40 cycles involving denaturation at 95°C for 15 s, annealing at 60°C for 20 s and elongation at 72°C for 20 s. The sequences of the specific sets of primer for p53, bax, bcl-2, caspase-3 and β-actin used in this study are given in our previous publication [30]. Expressions of selected genes were normalized to β-actin gene, which was used as an internal housekeeping control. All the real-time PCR experiments were performed in triplicate and data expressed as the mean of at least three independent experiments.

### Western blotting

HepG2 cells were cultured in 6-well plates and exposed to 10 µg/ml of CuO NPs for 24 h. The harvested cell pellets were lysed in RIPA lysis buffer [1X TBS (0.5 M Tris-HCl and 1.5 M NaCl) pH 7.4, 1% NP-40, 0.5% sodium deoxycholate, 0.1% SDS, 0.004% sodium azide] in the presence of a protease inhibitor [26]. The cell lysates were then analyzed for protein content using SDS-Page immunoblotting. The membrane was then probed with p53, bax, bcl-2, cleaved caspase-3 and β-actin antibodies to determine the expression of proteins. The β-actin was used as a loading control. Protein levels were also analyzed by desitometric analysis using AlphaEase TM FC StandAlone V.4.0.0 software. Results are expressed as a fold change over the control group.

### Protein estimation

The total protein content in cell extracts was estimated by the Bradford method [37] (Bradford 1976) using bovine serum albumin as the standard.

### Statistical analysis

All the data represented in this study are means ± SD of three identical experiments made in three replicate. Statistical significance was determined by one-way analysis of variance (ANOVA) followed by Dunnett's multiple comparison test. Significance was ascribed at p<0.05. All analyses were conducted using the Prism software package (GraphPad Software, Version 5.0).

## Results

### Physicochemical characterization of CuO nanoparticles

**Figure 1:**
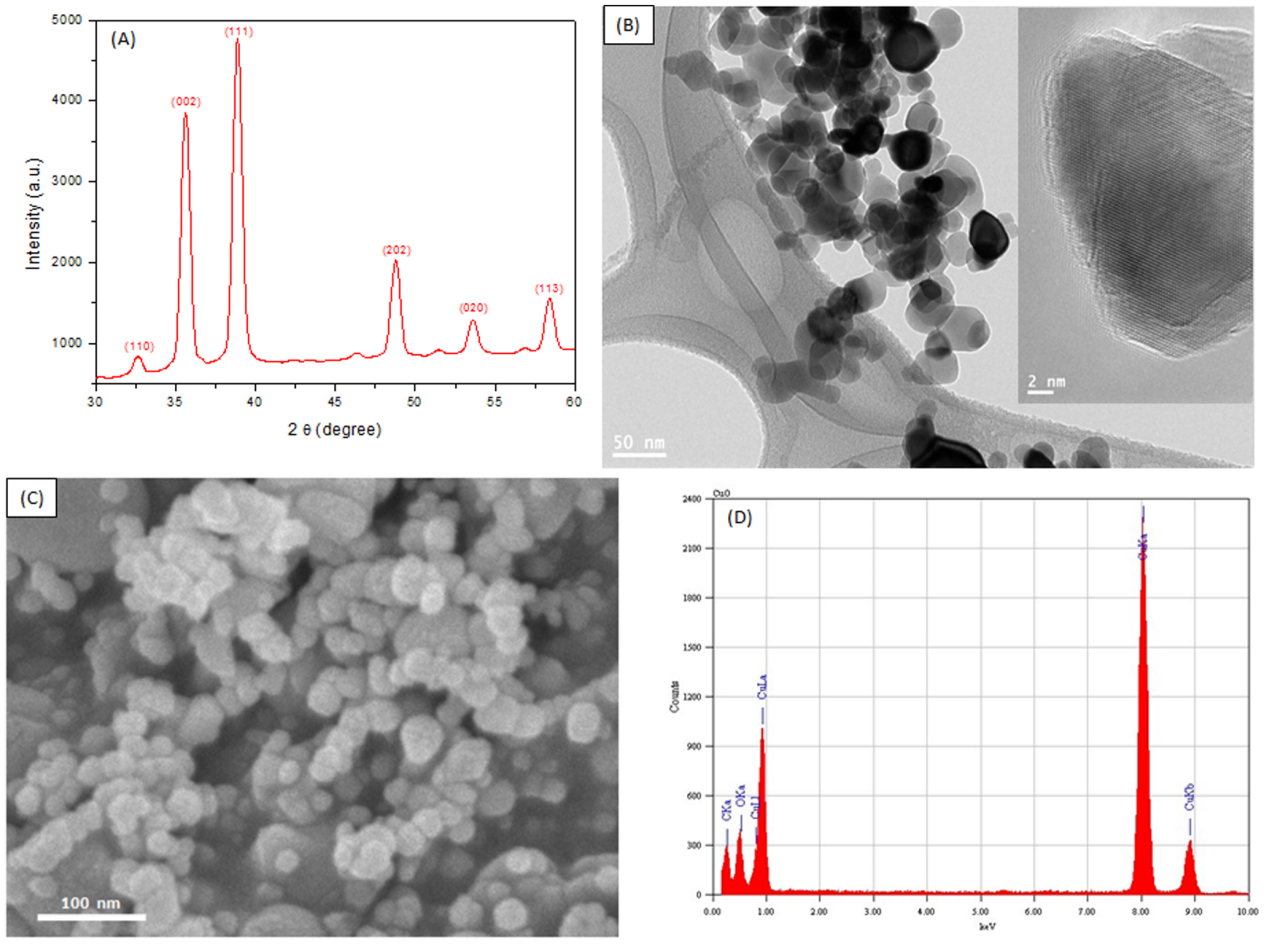
Characterization of CuO NPs. (A) XRD pattern, (C) FETEM image (inset with higher magnification), (C) FESEM image and (D) EDS spectrum.

**Figure 2:**
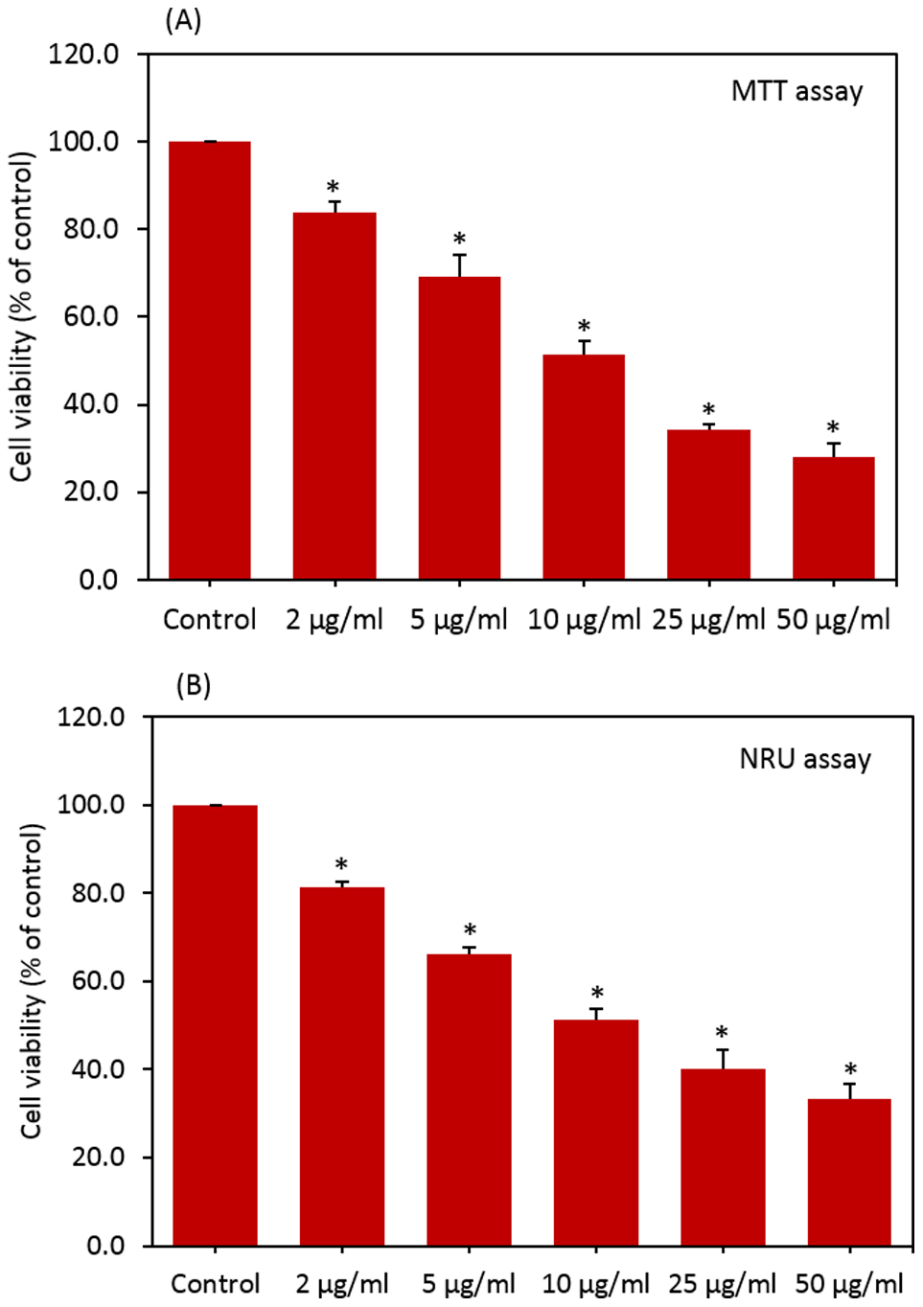
CuO NPs induced dose-dependent cytotoxicity in HepG2 cells. (A) MTT assay and (B) NRU assay. Data represented are mean ± SD of three identical experiments made in three replicate. *Statistically significant difference as compared to the controls (p<0.05 for each).

Fig. 1A shows the XRD pattern of prepared CuO NPs that clearly exhibits the crystalline nature of this material. The peaks at 2θ = 32.62°, 35.61°, 38.80°, 48.75°, 53.68° and 58.42 were assigned to (110), (002), (111), (202), (020) and (113) of CuO NPs. The crystallite size has been estimated from the XRD pattern using the Scherrer's equation [38]. The average crystallite size of CuO NPs was found to be around 22 nm. Fig. 2B and C show the typical FETEM and FESEM images of the CuO NPs, respectively. These pictures exhibit that the majority of the particles were spherical shaped with smooth surfaces. HRTEM (Fig. 2B inset image) also suggested the crystalline nature of CuO NPs. TEM average diameter was calculated from measuring over 100 particles in random fields of TEM view. The average TEM diameter of CuO NPs was also approximately 22 nm, supporting XRD results. EDS spectrum of CuO NPs is given in Fig. 2D. The EDS result shows that there are no other elemental impurities present in the prepared CuO NPs. The specific surface area of CuO NPs determined by BET was 34 m^2^/g. The average hydrodynamic size of CuO NPs in water and cell culture media determined by DLS was around 196 nm and 167 nm, respectively. Further, the zeta potential of CuO NPs in water and culture media was approximately −20 mV and −25 mV, respectively. The physicochemical characteristics of CuO NPs are listed in Table 1.

**Table 1:** Physicochemical properties of CuO NPs.

Parameters	Values (mean ± SD)
Average XRD size (nm)	22.24±3.67
Average TEM size (nm)	22.83±5.48
Surface area (m^2^/g)	34.62±2.11
Hydrodynamic size in distilled water (nm)	196.36±20.18
Hydrodynamic size in culture medium (nm)	167.48±18.69
Zeta potential distilled water (-mV)	20.36±1.34
Zeta potential culture medium (-mV)	25.41±2.25
Elemental impurities (by EDS spectrum)	Not detected

### Cytotoxic effects of CuO nanoparticles

HepG2 cells were exposed to CuO NPs at the concentrations of 0, 2, 5, 10, 25 and 50 µg/ml for 24 h and cytotoxicity was determined using MTT and NRU assays. Results showed that CuO NPs significantly decreased the cell viability in dose-dependent manner. In MTT assay cell viability decreased to 83%, 69%, 52%, 34% and 28% when cells exposed to CuO NPs at the concentrations of 2, 5, 10, 25 and 50 µg/ml, respectively (Fig. 2A). Fig. 2B shows the results of cell viability obtained by NRU assay. In NRU assay cell viability decreased to 81%, 66%, 51%, 40% and 33% when cells exposed to CuO NPs at the concentrations of 2, 5, 10, 25 and 50 µg/ml, respectively. In agreement with cell viability data phase contrast microscopy results also showed that lowering of cell density and rounding of cells in dose-dependent manner due to CuO NPs exposure (Fig. S1 in Supporting Information S1).

Moreover, measurement of Cu^2+^ released in cell culture medium suggested that Cu^2+^ released from CuO NPs were not involved in the toxicity induced by these nanoparticles on HepG2 cells (Fig S2 in Supporting Information S1).

### Toxicity of CuO nanoparticlse was mediated through oxidative stress

**Figure 3:**
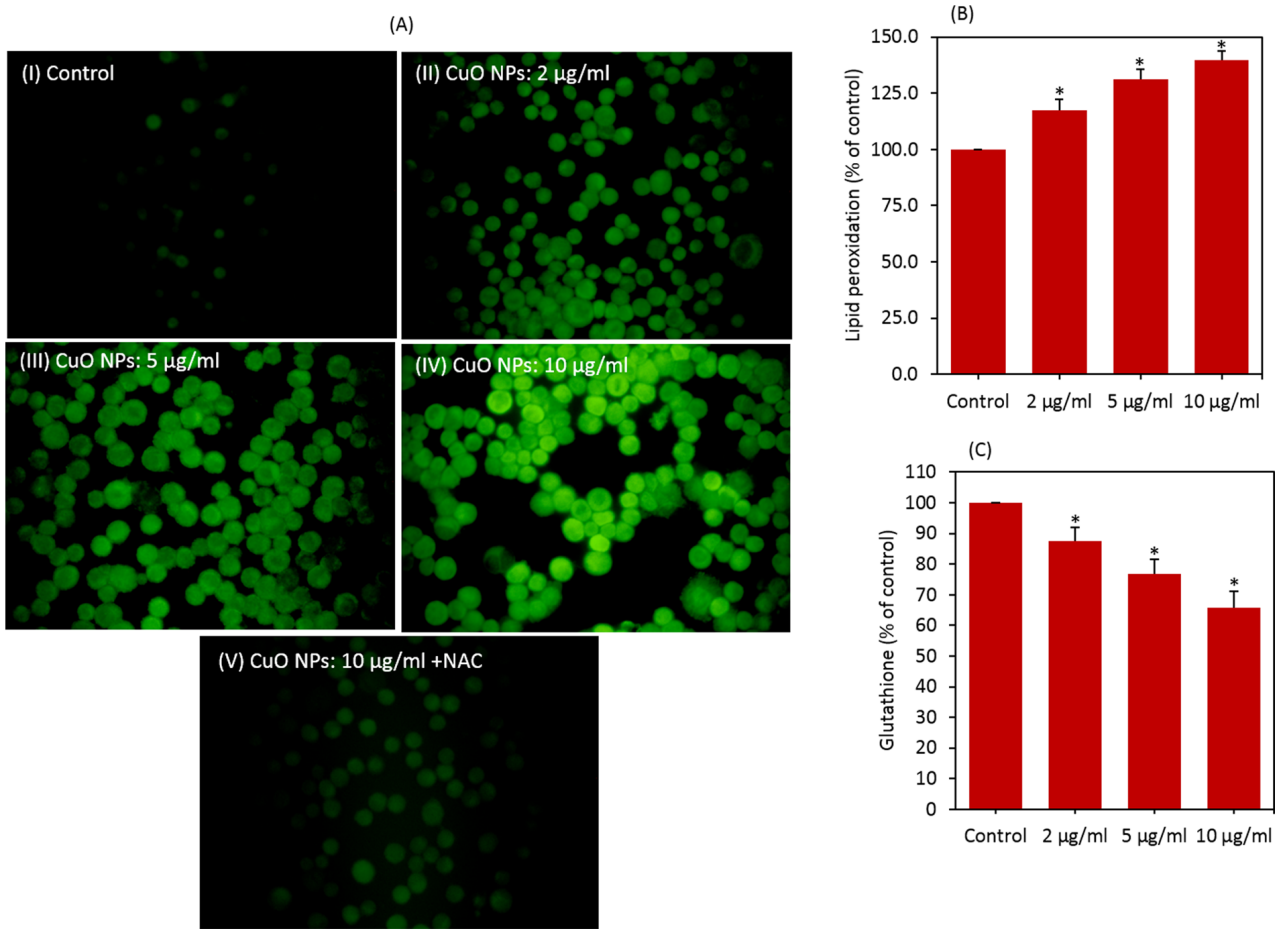
CuO NPs induced dose-dependent oxidative stress in HepG2 cells. (A) ROS level in HepG2 cells treated with CuO NPs in presence or absence of NAC, (B) lipid peroxidation and (C) glutathione level. Data represented are mean ± SD of three identical experiments made in three replicate. *Statistically significant difference as compared to the controls (p<0.05 for each).

We examined the effect of CuO NPs on ROS generation in the presence or absence of the anti-oxidant N-acetyl-cystein (NAC). Fluorescent microscopy data revealed that CuO NPs (2–10 µg/ml) induced the intracellular production of ROS in dose-dependent manner (Fig. 3A). We further observed that co-exposure of NAC effectively prevented the ROS generation induced by 10 µg/ml of CuO NPs. ROS level was reduced up to control level for CuO NPs in the presence of NAC.

We also determined the membrane lipid peroxidation (LPO) and intracellular glutathione (GSH) levels in HepG2 cells exposed to CuO NPs (2–10 µg/ml) for 24 h. MDA level, an end product of membrane LPO was significantly higher in cells exposed to CuO NPs in the concentration range of 2–10 µg/ml (p<0.05 for each) (Fig. 3B). Fig. 3C shows that CuO NPs significantly reduced the intracellular level of GSH in a dose-dependent manner (p<0.05 for each).

**Figure 4:**
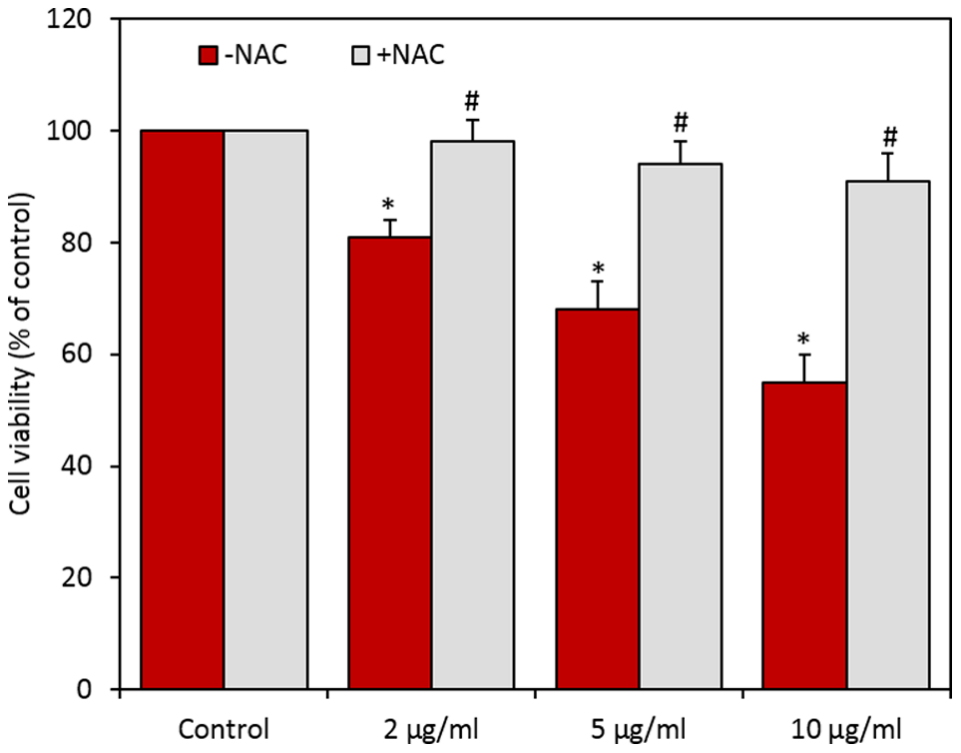
N-acetyl-cystein (NAC) significantly preserved the viability (MTT assay) of HepG2 cells caused by CuO NPs. Data represented are mean ± SD of three identical experiments made in three replicate. *Statistically significant difference in percentage of cells as compared to the controls (p<0.05). ^#^Significant inhibitory effect of NAC on cell viability reduction (p<0.05).

In order to investigate whether oxidative stress could play a role in the cytotoxicity of CuO NPs, HepG2 cells were exposed to CuO NPs in the presence of the NAC. Results showed that NAC abolished almost fully the harmful effect of CuO NPs at all concentrations studied (Fig. 4)

### CuO nanoparticles decreased the mitochondrial membrane potential

**Figure 5:**
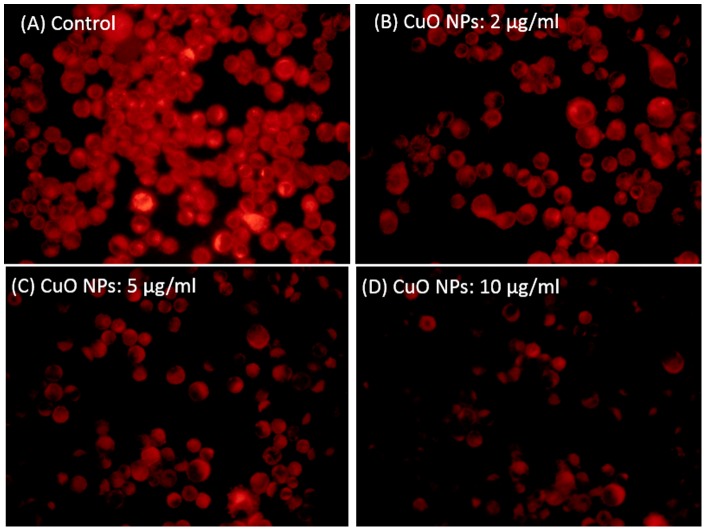
CuO NPs induced dose-dependent mitochondrial membrane potential in HepG2 cells.

It is well known that during apoptosis the mitochondrial membrane potential (MMP) decreases [27]. The effect of CuO NPs on mitochondrial membrane potential (MMP) was evaluated in HepG2 cells. Cells were exposed to CuO NPs (2–10 µg/ml) for 24 h and assayed for Rhodamine 123 uptake using fluorescence microscope. The brightness of the fluorescent intensity was reduced in cells exposed to CuO NPs that indicates a significant reduction of mitochondrial membrane potential in HepG2 cells (Fig. 5).

### CuO nanoparticles induced apoptosis

**Figure 6:**
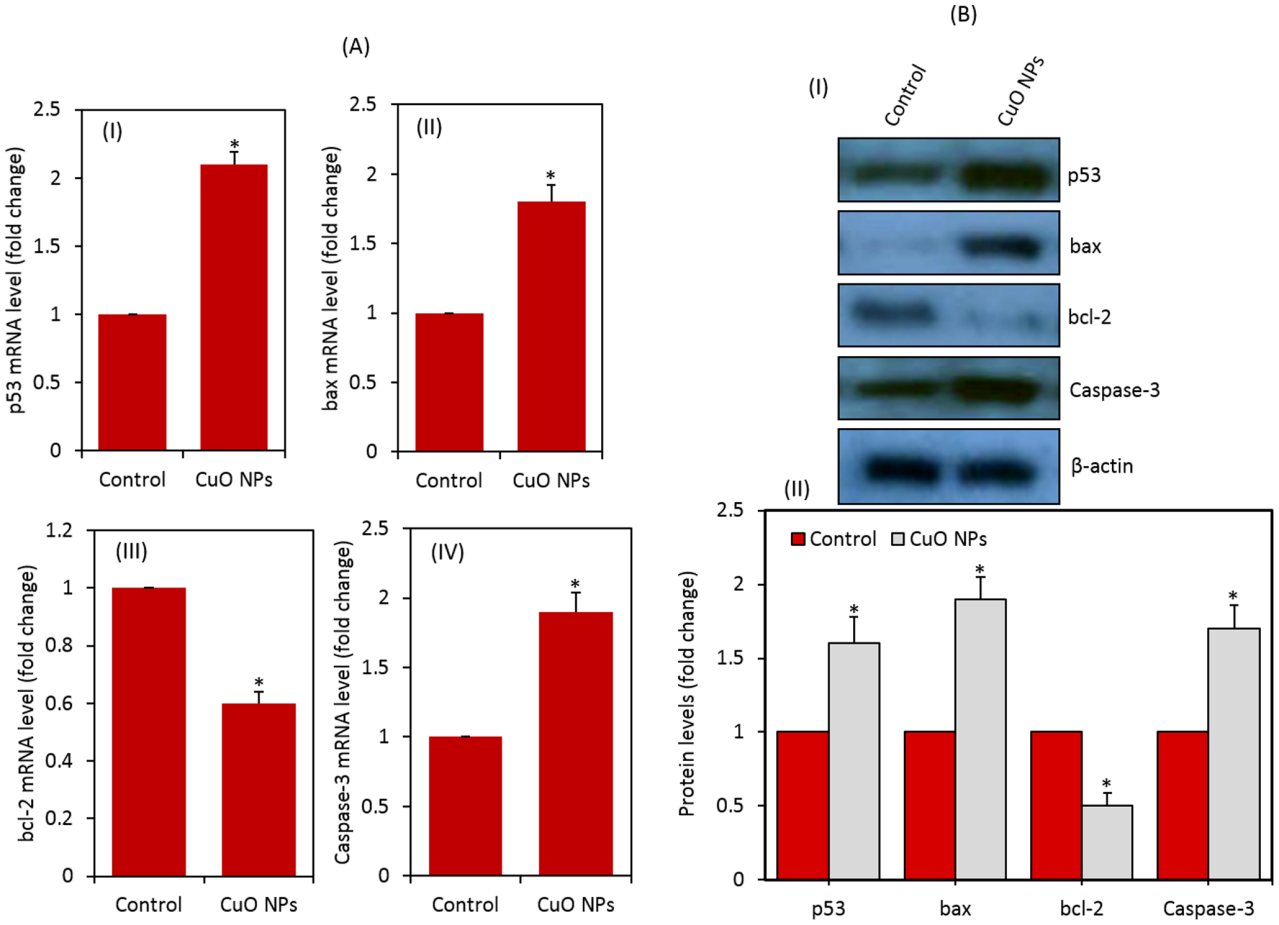
CuO NPs induced apoptosis in HepG2 cells. (A) Quantitative real-time PCR analysis of mRNA levels of apoptotic genes in HepG2 cells exposed to CuO NPs. (I) p53, (II) bax, (III) bcl-2 and (IV) caspase-3. Data represented are mean ± SD of three identical experiments made in three replicate. *Statistically significant difference as compared to the controls (p<0.05 for each). (B) Western blot analysis of protein levels of apoptotic genes in HepG2 cells treated with CuO NPs. (I) Immunoblot images of p53, bax, bcl-2 and bax proteins. (II) Protein levels were also analyzed by desitometric analysis using AlphaEase TM FC StandAlone V.4.0.0 software. Results are expressed as a fold change over the control group. *Statistically significant difference as compared to the controls (p<0.05 for each).

Quantitative real-time PCR was used to analyze the mRNA levels of apoptotic genes (p53, bax, bcl-2, and caspase-3) in HepG2 cells exposed to CuO NPs at the concentration of 10 μg/ml for 24 h. Results showed that CuO NPs significantly altered the expression levels of mRNA of these genes in HepG2 cells. The mRNA expression level of tumor suppressor gene p53 and apoptotic genes bax & caspase-3 were significantly up-regulated while the expression of anti-apoptotic gene bcl-2 was significantly down-regulated in CuO NPs treated cells as compared to controls (Fig. 6A) (p<0.05 for each).

To confirm the quantitative real-time PCR results, we further examined the protein expression levels of these genes in CuO NPs exposed HepG2 cells using Western blotting. Similar to mRNA results, the protein levels of p53, bax and cleaved caspase-3 were significantly up-regulated while the expression of bcl-2 was significantly down-regulated in CuO NPs treated cells (p<0.05 for each) (Fig. 6B).

## Discussion

Toxicity of CuO NPs has been considered as a serious limitation for their implementation in new applications and prior mechanistic toxicological characterization of this material is needed [12], [13]. The present study demonstrates the cytotoxicity, oxidative stress and apoptosis response of CuO NPs on human hepatocellular carcinoma HepG2 cells. Our data also revealed that the mode of cell death was apoptosis which was mediated by the ROS triggered mitochondria pathway as evidenced by decrease in MMP along with modulation of bax, bcl-2 and cleaved caspase-9 proteins expression.

Physicochemical characterization of nano-scale materials is essential in nanotoxicity research for better interpretation of results [28], [39], [40]. Characterization of CuO NPs was done using a combination of XRD, TEM, EDS, DLS and BET techniques in order to provide clear insight into crystalline nature, morphology, particle size, surface property and the chemical composition. Our XRD results confirm the crystalline nature of CuO NPs. SEM and TEM showed that nanoparticles were spherical in shape with a smooth surface and had an average diameter of 22 nm. EDS data indicated that synthesized CuO NPs were highly pure with no traces of impurities. Agglomeration and stability of nanoparticles in aqueous suspension are major challenges nanotoxicology [26], [41]. Once the nanomaterials were introduced to aqueous media, the sizes changed to approximately five to ten times of the primary size. We found the same results in case of CuO NPs (Table 1). The higher size of nanoparticles in aqueous suspension as compared to TEM and XRD might be due to the tendency of particles to agglomerate in aqueous state. This finding is supported by other investigators [42], [43] and has been discussed in our previous publications [26], [41]. In brief, DLS measures Brownian motion and subsequent size distribution of an ensemble collection of nanoparticles in suspension gives a mean hydrodynamic diameter that is usually larger than TEM diameter because it represents a dried layer of nanoparticles on a TEM grid. During DLS measurement, there is a tendency of nanoparticles to agglomerate in aqueous state thereby giving the size of clustered particles rather than individual particles [27]. Moreover, mean hydrodynamic size of CuO NPs in complete cell culture medium was found to be about slightly smaller than that in distilled water. Similar drop in the hydrodynamic size of nanoparticles in culture medium was reported by other researchers [44], [45], suggesting that salts and proteins could help in nanoparticle dispersion in an aqueous environment. The tendency of particles to form aggregates depends on the surface charge. The CuO NPs charge, determined as zeta potential was −20 and −25 mV for distilled water and cell culture media, respectively.

Oxidative stress has been suggested to play an important role in the toxicity mechanisms of nanoparticles [46], [47]. This has been attributed due to their small size and large surface area which is generally thought to generate ROS. ROS such as superoxide anion (O2^−^), hydroxyl radical (HO^•^) and hydrogen peroxide (H_2_O_2_) elicit a variety of physiological and cellular events including inflammation, DNA damage and apoptosis [39], [48], [49]. There has been an increase in biochemical and clinical evidences that indicate the involvement of ROS and oxidative stress in various diseases including cancer [50], [51]. In previous study, we found that CuO NPs were able to induce ROS mediated cytotoxicity and genotoxicity in A549 cells [10]. In the present study, ROS and lipid peroxidation levels were significantly higher while the antioxidant GSH level was significantly lower in HepG2 cells exposed to CuO NPs. Moreover, antioxidant NAC reduced cell death drastically indicating that oxidative stress plays an important role in CuO NPs induced cytotoxicity.

ROS has been suggested to be signaling molecule for the initiation and execution of the apoptotic cell death program [52]. The production of ROS, in particular, has also been associated with programmed cell death in many conditions such as stroke, inflammation, ischemia, lung edema and neuro-degeneration [53], [54]. In the present study, we observed that the expressions of both mRNA and protein levels of tumor suppressor gene p53 and apoptotic genes (bax and cleaved caspase-3) were up-regulated while the expression of anti-apoptotic gene bcl-2 was down-regulated in HepG2 cells treated with CuO NPs. It has been suggested that bax is up-regulated by p53 [55]. Since an increase in bax expression was observed, the role of p53 in the up-regulation of bax upon CuO NPs exposure can be postulated. The insertion of bax into the mitochondrial membrane possibly leads to p53-mediated apoptosis [55]. Caspases are activated during apoptosis in many cells and are known to play a vital role in both initiation and execution of apoptosis. It was reported that activated caspase-3 (cleaved caspase-3) is essential for cellular DNA damage and apoptosis [56]. Taken together, up-regulation of p53 leads to activation of pro-apoptotic members of bcl-2 family, such as bax induces permeabilization of the outer mitochondrial membrane, which releases soluble proteins from the intermembrane space into the cytosol, where they promote caspase activation [57]. The best studied of these proteins is cytochrome c, which binds to apoptosis protease activating factor-1 (Apaf-1) and leads to the assembly of an apoptosome complex. This apoptosome can bind procaspase-9 and cause its auto-activation through a conformational change. Once initiated caspase-9 goes on to activate caspase-3 (effector caspase), which cleaves substrates at aspartate residues and activation of this proteolytic activity appears to be an event in apoptosis [25]. Results of the present study are supported by our previous studies where ZnO NPs nanoparticles significantly up-regulated of p53 and bax genes along with the down-regulated proapototic gene bcl-2 in A549 and HepG2 cells [41], [49].

## Conclusion

CuO NPs were found to induce cytotoxicity in HepG2 cells in dose-dependent manner, which was likely to be mediated through ROS. Tumor suppressor gene p53 and apoptotic gene caspase-3 were up-regulated due to CuO NPs exposure. Decrease in MMP with a concomitant increase in the expression of bax/bcl2 ratio suggested that CuO NPs induced apoptosis in HepG2 cells through mitochondrial pathway. Our study provided valuable insights into the possible mechanism of liver toxicity caused by CuO NPs at *in vitro* level. Our short-term exposure study of high level induction of apoptotic response of CuO NPs will need to be further investigated to determine whether long-term exposure consequences may exist for CuO NPs application.

## Supporting Information

Supporting Information S1 Morphology of HepG2 cells exposed to different concentrations of CuO NPs for 24 h. (A) control, (B) 2 µg/ml, (C) 5 µg/ml, (D) 10 µg/ml, (E) 25 µg/ml and (F) 50 µg/ml. **Figure S2**, Effect of dissolved Cu^2+^ on the viability and morphology of HepG2 cells. (A) MTT assay and (B) NRU assay. Data represented are mean ± SD of three identical experiments made in three replicate. *Statistically significant difference as compared to the controls (p<0.05 for each). (C) Morphology of HepG2 cells exposed to Cu^2+^ at the concentration of 5.0 µg/mL for 24 h.
(DOCX)

## References

[1] Jiang LC, Zhang WD (2010) A highly sensitive nonenzymatic glucose sensor based on CuO nanoparticles-modified carbon nanotube electrode. Biosens Bioelectron 25: 1402–1407. 19942424 10.1016/j.bios.2009.10.038

[2] Song MJ, Hwang SW, Whang D (2010) Non-enzymatic electrochemical CuO nanoflowers sensor for hydrogen peroxide detection. Talanta 80: 1648–1652. 20152391 10.1016/j.talanta.2009.09.061

[3] Dastjerdi R, Montazer M (2010) A review on the application of inorganic nanostructured materials in the modification of textiles: focus on antimicrobial properties. Colloids Surf B Biointerfaces 79: 5–18. 20417070 10.1016/j.colsurfb.2010.03.029

[4] Delgado K, Quijada R, Palma R, Palza H (2011) Polypropylene with embedded copper metal or copper oxide nanoparticles as a novel plastic antimicrobial agent. Lett Appl Microbiol 53: 50–54. 21535046 10.1111/j.1472-765X.2011.03069.x

[5] Mortimer M, Kasemets K, Kahru A (2010) Toxicity of ZnO and CuO nanoparticles to ciliated protozoa *Tetrahymena thermophila* . Toxicology 269: 182–189. 19622384 10.1016/j.tox.2009.07.007

[6] Zhao J, Wang Z, Liu X, Xie X, Zhang K, et al (2011) Distribution of CuO nanoparticles in juvenile carp (Cyprinus carpio) and their potential toxicity. J Hazard Mater 197: 304–310. 22014442 10.1016/j.jhazmat.2011.09.094

[7] Pradhan A, Seena S, Pascoal C, Cássio F (2012) Copper oxide nanoparticles can induce toxicity to the freshwater shredder *Allogamus ligonifer* . Chemosphere 89: 1142–1150. 22749936 10.1016/j.chemosphere.2012.06.001

[8] Gomes T, Pereira CG, Cardoso C, Pinheiro JP, Cancio I, et al (2012) Accumulation and toxicity of copper oxide nanoparticles in the digestive gland of *Mytilus galloprovincialis* . Aquatic Toxicol 118–119: 72–79. 10.1016/j.aquatox.2012.03.017 22522170

[9] Fahmy B, Cormier SA (2009) Copper oxide nanoparticles induce oxidative stress and cytotoxicity in airway epithelial cells. Toxicol In Vitro 23: 1365–1371. 19699289 10.1016/j.tiv.2009.08.005 PMC2756312

[10] Ahamed M, Siddiqui MA, Akhtar MJ, Ahmad I, Pant AB, et al (2010) Genotoxic potential of copper oxide nanoparticles in human lung epithelial cells. Biochem Biophys Res Commun 396: 578–583. 20447378 10.1016/j.bbrc.2010.04.156

[11] Sun J, Wang S, Zhao D, Hun FH, Weng L, et al (2011) Cytotoxicity, permeability, and inflammation of metal oxide nanoparticles in human cardiac microvascular endothelial cells: cytotoxicity, permeability, and inflammation of metal oxide nanoparticles. Cell Biol Toxicol 27: 333–342. 21681618 10.1007/s10565-011-9191-9

[12] Xu J, Li Z, Xu P, Xiao L, Yang Z (2012) Nanosized copper oxide induces apoptosis through oxidative stress in podocytes. Arch Toxicol (DOI 10.1007/s00204–012–0925–0). 10.1007/s00204-012-0925-0 22903339

[13] Perreault F, Melegari SP, Costa CH, Rossetto A-F, Popovic R, et al (2012) Genotoxic effects of copper oxide nanoparticles in Neuro 2A cell cultures. Sci Total Environ 441: 117–124. 23137976 10.1016/j.scitotenv.2012.09.065

[14] Nishimori H, Kondoh M, Isoda K, Tsunoda S, Tsutsumi Y, et al (2009) Silica nanoparticles as hepatotoxicants. Eur J Pharm Biopharm 72: 496–501. 19232391 10.1016/j.ejpb.2009.02.005

[15] Xie G, Sun J, Zhong G, Shi L, Zhang D (2010) Biodistribution and toxicity of intravenously administered silica nanoparticles in mice. Arch Toxicol 84: 183–190. 19936708 10.1007/s00204-009-0488-x

[16] Wang Y, Aker WG, Hwang HM, Yedjou CG, Yu H, et al (2011) A study of the mechanism of in vitro cytotoxicity of metal oxide nanoparticles using catfish primary hepatocytes and human HepG2 cells. Sci Total Environ 409: 4753–4762. 21851965 10.1016/j.scitotenv.2011.07.039 PMC3185176

[17] Piret JP, Jacques D, Audinot JN, Mejia J, Boilan E, et al (2012) Copper (II) oxide nanoparticles penetrate into HepG2 cells, exert cytotoxicity via oxidative stress and induce pro-inflammatory response. Nanoscale 4: 7168–7184. 23070296 10.1039/c2nr31785k

[18] Ashkenazi A (2002) Targeting death and decoy receptors of the tumor-necrosis factor superfamily. Nat Rev Cancer 2: 420–430. 12189384 10.1038/nrc821

[19] Liu Y, Martin M (2001) P53 protein at the hub of cellular DNA damage response pathways through sequence-specific and non-sequence-specific DNA binding. Carcinogenesis 22: 851–860. 11375889 10.1093/carcin/22.6.851

[20] Ahamed M, Karns M, Goodson M, Rowe J, Hussain S, et al (2008) DNA damage response to different surface chemistry of silver nanoparticles in mammalian cells. Toxicol Appl Pharmacol 233: 404–410. 18930072 10.1016/j.taap.2008.09.015

[21] Li T, Kon N, Jiang L, Tan M, Ludwig T, et al (2012) Tumor suppression in the absence of p53-mediated cell-cycle arrest, apoptosis, and senescence. Cell 149: 1269–1283. 22682249 10.1016/j.cell.2012.04.026 PMC3688046

[22] Reed JC (2006) Proapoptotic multidomain bcl-2/bax family proteins: mechanisms, physiological roles and therapeutic opportunities. Cell Death Different 13: 1378–1386. 10.1038/sj.cdd.4401975 16729025

[23] Chougule M, Patel AR, Sachdeva P, Jackson T, Singh M (2011) Anticancer activity of Noscapine, an opioid alkaloid in combination with cisplatin in human non-small cell lung cancer. Lung Cancer 71: 271–282. 20674069 10.1016/j.lungcan.2010.06.002 PMC3094914

[24] Gao C, Wang AY (2009) Significance of increased apoptosis and bax expression in human small intestinal adenocarcinoma. J Histochem Cytochem 257: 1139–1148. 10.1369/jhc.2009.954446 PMC2778087 19729672

[25] Youle RJ, Strasser A (2008) The BCL-2 protein family: opposing activities that mediate cell death. Nat Rev Mol Cell Biol 9: 47–59. 18097445 10.1038/nrm2308

[26] Ahmad J, Ahamed M, Akhtar MJ, Alrokayan SA, Siddiqui MA, et al (2012) Apoptosis induction by amorphous silica nanoparticles mediated through reactive oxygen species generation in human liver cell line HepG2. Toxicol Appl Pharmacol 259: 160–168. 22245848 10.1016/j.taap.2011.12.020

[27] Sharma V, Anderson D, Dhawan A (2012) Zinc oxide nanoparticles induce oxidative DNA damage and ROS-triggered mitochondria mediated apoptosis in human liver cells (HepG2). Apoptosis 17: 852–870. 22395444 10.1007/s10495-012-0705-6

[28] Murdock RC, Braydich-Stolle L, Schrand AM, Schlager JJ, Hussain SM (2008) Characterization of nanomaterial dispersion in solution prior to in vitro exposure using dynamic light scattering technique. Toxicol Sci 101: 239–253. 17872897 10.1093/toxsci/kfm240

[29] Mossman T (1983) Rapid colorimetric assay for cellular growth and survival: application to proliferation and cytotoxicity assays. J Immunol Methods 65: 55–63. 6606682 10.1016/0022-1759(83)90303-4

[30] Ahamed M, Akhtar MJ, Siddiqui MA, Ahmad J, Musarrat J, et al (2011a) Oxidative stress mediated apoptosis induced by nickel ferrite nanoparticles in cultured A549 cells. Toxicology 283: 101–118. 21382431 10.1016/j.tox.2011.02.010

[31] Borenfreund E, Puerner JA (1984) A simple quantitative procedure using monolayer cultures for cytotoxicity assays. J Tissue Cult Method 9: 7–9.

[32] Zhang Y, Jiang L, Jiang L, Geng C, Li L, et al (2011) Possible involvement of oxidative stress in potassium bromate-induced genotoxicity in human HepG2 cells. Chem Biol Int 189: 186–191. 10.1016/j.cbi.2010.12.011 21182833

[33] Ohkawa H, Ohishi N, Yagi K (1979) Assay for lipid peroxides in animal tissues by thiobarbituric acid reaction. Anal Biochem 95: 351–358. 36810 10.1016/0003-2697(79)90738-3

[34] Ellman GI (1959) Tissue sulfhydryl groups. Arch Biochem Biophys 82: 70–77. 13650640 10.1016/0003-9861(59)90090-6

[35] Wang H, Joseph JA (1999) Quantifying cellular oxidative stress by dichlorofluorescein assay using microplate reader. Free Radic Biol Med 27: 612–661. 10490282 10.1016/s0891-5849(99)00107-0

[36] Siddiqui MA, Kashyap MP, Kumar V, Al-Khedhairy AA, Musarrat J, et al (2010) Protective potential of trans-resveratrol against 4-hydroxynonenal induced damage in PC12 cells. Toxicol In Vitro 24: 1592–1598. 20600804 10.1016/j.tiv.2010.06.008

[37] Bradford MM (1976) A rapid and sensitive for the quantitation of microgram quantities of protein utilizing the principle of protein-dye binding. Anal Biochem 72: 248–254. 942051 10.1016/0003-2697(76)90527-3

[38] Patterson AL (1939) The Scherrer formula for x-ray particle size determination. Phys Rev 56: 978–982.

[39] Nel A, Xia T, Madler L, Li N (2006) Toxic potential of materials at the nanolevel. Science 311: 622–627. 16456071 10.1126/science.1114397

[40] Yu KO, Grabinski CM, Schrand AM, Murdock RC, Wang W, et al (2009) Toxicity of amorphous silica nanoparticles in mouse keratinocytes. J Nanopart Res 11: 15–24.

[41] Akhtar MJ, Ahamed M, Kumar S, Ahmad J, Khan MAM, et al (2012) Zinc oxide nanoparticles selectively induces apoptosis in cancer cells through reactive oxygen species: Int J Nanomed. 7: 845–857. 10.2147/IJN.S29129 PMC3289443 22393286

[42] Bai W, Zhang Z, Tian W, He X, Ma Y, et al (2009) Toxicity of zinc oxide nanoparticles to zebrafish embryo: A physicochemical study of toxicity mechanism. J Nanopart Res 12: 1645–1654.

[43] Sharma V, Shukla RK, Saxena N, Parmar D, Das M, et al (2009) DNA damaging potential of zinc oxide nanoparticles in human epidermal cells. Toxicol Lett 185: 211–218. 19382294 10.1016/j.toxlet.2009.01.008

[44] Xia T, Kovochich M, Liong M, Mädler L, Gilbert B, et al (2006) Comparison of the mechanism of toxicity of zinc oxide and cerium oxide nanoparticles based on dissolution and oxidative stress properties. ACS Nano 2: 2121–2134. 10.1021/nn800511k PMC3959800 19206459

[45] Ng KW, Khoo SK, Heng BC, Setyawati MI, Tan EC, et al (2011) The role of the tumor suppressor p53 pathway in the cellular DNA damage response to zinc oxide nanoparticles. Biomaterials 32: 8218–8225. 21807406 10.1016/j.biomaterials.2011.07.036

[46] Wise JP, Goodale BC, Wise SS (2010) Silver nanospheres are cytotoxic and genotoxic to fish cells. Aquatic Toxicol 97: 34–41. 10.1016/j.aquatox.2009.11.016 PMC4526150 20060603

[47] Siddiqui MA, Ahamed M, Ahmad J, Khan MAM, Musarrat J, et al (2012) Nickel oxide nanoparticles induce cytotoxicity, oxidative stress and apoptosis in cultured human cells that is abrogated by the dietary antioxidant curcumin. Food Chem Toxicol 50: 641–47. 22273695 10.1016/j.fct.2012.01.017

[48] Asharani PV, Mun GK, Hande MP, Valiyaveettil S (2009) Cytotoxicity and genotoxicity of silver nanoparticles in human cells. ACS Nano 3: 279–290. 19236062 10.1021/nn800596w

[49] Ahamed M, Akhtar MJ, Raja M, Ahmad I, Siddiqui MKJ, et al (2011b) Zinc oxide nanorod induced apoptosis via p53, bax/bcl-2 and survivin pathways in human lung cancer cells: Role of oxidative stress. Nanomedicine: NBM 7: 904–913. 10.1016/j.nano.2011.04.011 21664489

[50] Benz CC, Yau C (2008) Ageing, oxidative stress and cancer: paradigms in parallax. Nat Rev Cancer 8: 875–879. 18948997 10.1038/nrc2522 PMC2603471

[51] Jomova K, Valko M (2011) Advances in metal-induced oxidative stress and human disease. Toxicology 283: 65–87. 21414382 10.1016/j.tox.2011.03.001

[52] Ott M, Gogvadze V, Orrenius S, Zhivotovsky B (2007) Mitochondria, oxidative stress and cell death. Apoptosis 12: 913–922. 17453160 10.1007/s10495-007-0756-2

[53] Kannan K, Jain SK (2000) Oxidative stress and apoptosis. Pathophysiology 7: 153–163. 10996508 10.1016/s0928-4680(00)00053-5

[54] Bai J, Meng Z (2005) Effects of sulfur dioxide on apoptosis-related gene expressions in lungs from rats. Regul Toxicol Pharmacol 43: 272–279. 16256253 10.1016/j.yrtph.2005.09.002

[55] Gopinath P, Gogoi SK, Sanpui P, Paul A, Chattopadhyay A, et al (2010) Signaling gene cascade in silver nanoparticle induced apoptosis. Colloids Surf B 77: 240–245. 10.1016/j.colsurfb.2010.01.033 20197232

[56] Janicke RU, Sprengart ML, Wati MR, Porter AG (1998) Caspase-3 is required for DNA fragmentation and morphological changes associated with apoptosis. J Biol Chem 273: 9357–9360. 9545256 10.1074/jbc.273.16.9357

[57] Fuentes-Prior P, Salvesen GS (2004) The protein structures that shape caspase activity, specificity, activation and inhibition. J Biochem 384: 201–232. 10.1042/BJ20041142 PMC1134104 15450003

