# Supplementary material for: Copper Oxide Nanoparticles Induced Mitochondria Mediated Apoptosis in Human Hepatocarcinoma Cells
Source: PLoS One. 2013 Aug 5;8(8):e69534. doi: 10.1371/journal.pone.0069534 (PMC3734287; doi:10.1371/journal.pone.0069534)
Supplement: Supporting Information S1 — Morphology of HepG2 cells exposed to different concentrations of CuO NPs for 24 h. (A) control, (B) 2 µg/ml, (C) 5 µg/ml, (D) 10 µg/ml, (E) 25 µg/ml and (F) 50 µg/ml. Figure S2, Effect of dissolved Cu2+ on the viability and morphology of HepG2 cells. (A) MTT assay and (B) NRU assay. Data represented are mean ± SD of three identical experiments made in three replicate. *Statistically significant difference as compared to the controls (p<0.05 for each). (C) Morphology of HepG2 cells exposed to Cu2+ at the concentration of 5.0 µg/mL for 24 h. (DOCX) [file pone.0069534.s001.docx]

**Supporting Information**

**Effect of CuO NPs on morphology of HepG2 cells**

Morphology of HepG2 cells was examined following exposure to different concentrations of CuO NPs by using a phase-contrast microscope (Leica DMIL, Germany). Figure S1 shows the comparative morphology of control and CuO NPs treated HepG2 cells. A significant lowering of cell density and rounding of cells were observed in dose-dependent manner. Morphological changes in cells were visible after 5 μg/mL CuO NPs exposure in 24 h (Fig. S1 C). Most of the cells become spherical and detached from the surface at the concentration of 25 and 50 µg/ml for 24 h (Fig. S1 E & F).

**Analysis of dissolution of CuO nanoparticles and exposure of cells with the same**

To determine whether Cu^2+^ released from CuO NPs suspension may play a role in cellular toxicity, the released Cu^2+^ concentrations were measured in all the culture media collected from CuO NPs treatment. Culture media were taken from CuO NPs treated cells immediately at the end of exposure and centrifuged at 30000 g for 30 minutes. After centrifugation, the released Cu2+ concentrations in the supernatants were detected by graphite furnace atomic absorption spectrometry (GFAA). Moreover, to compare the toxic effects of released Cu^2+^ from CuO NPs suspensions, another exposure regime with soluble Cu^2+^ was performed. In that case, CuO NPs suspension was replaced by Cu^2+^ solutions (0.2, 0.5, 1, 2.5 and 5 µg/ml) which were dosed by adding the required mass of copper chloride (CuCl2, ≥99.995%, Sigma-Aldrich) to the culture medium. Criteria for the selection of Cu^2+^ concentrations are explained in the results section.

**Dissolution of CuO NPs in to Cu^2+^ and effects of soluble Cu^2+^ on HepG2 cells**

To determine whether our observed cytotoxicity could be attributed to the released Cu^2+^, we analyzed the level of Cu^2+^ released from CuO NPs suspension and tested the effect of this defined concentration of Cu^2+^ in HepG2 cells. The released Cu^2+^ concentrations in the CuO NPs suspension at the end of exposure were measured, and the highest dissolution of 4.97±0.36 μg/ml released Cu^2+^ was found in 50 μg/ml CuO NPs suspension. Furthermore, the released Cu^2+^ concentrations in 2, 5, 10, and 25 μg/ml CuO NPs treatments were 0.21±0.02, 0.49±0.03, 1.02± 0.08 and 2.49±0.19 μg/ml, respectively. Our data on the dissolution of CuO NPs in aqueous state are in agreement with other recent studies. Thus, we examined the toxic effect of 0.2, 0.5, 1, 2.5 and 5 μg/ml of soluble Cu^2+^ in HepG2 cells and found that Cu^2+^ caused no apparent effect on cell viability (**Figure S2 A & B**). Microscopy results also showed that soluble Cu^2+^ did not produce the adverse effect on the morphology of HepG2 cells up to the 5 µg/ml dose (**Figure S2 C**). These results suggest that toxicity observed by CuO NPs (in the concentration range 2-50 μg/ml) was solely due to CuO NPs, not soluble Cu^2+^.

**Supporting Information Legends**

**Figure S1.** Morphology of HepG2 cells exposed to different concentrations of CuO NPs for 24 h. (A) control, (B) 2 µg/ml, (C) 5 µg/ml, (D) 10 µg/ml, (E) 25 µg/ml and (F) 50 µg/ml.

**Figure S2.** Effect of dissolved Cu^2+^ on the viability and morphology of HepG2 cells. (A) MTT assay and (B) NRU assay. Data represented are mean±SD of three identical experiments made in three replicate. *Statistically significant difference as compared to the controls (p<0.05 for each). (C) Morphology of HepG2 cells exposed to Cu^2+^ at the concentration of 5.0 µg/mL for 24 h.
